# Regulation of HBEGF by Micro-RNA for Survival of Developing Human Trophoblast Cells

**DOI:** 10.1371/journal.pone.0163913

**Published:** 2016-10-04

**Authors:** Chandni V. Jain, Philip Jessmon, Brian A. Kilburn, Meritxell Jodar, Edward Sendler, Stephen A. Krawetz, D. Randall Armant

**Affiliations:** 1 Department of Physiology, Wayne State University School of Medicine, Detroit, Michigan, United States of America; 2 Department of Obstetrics and Gynecology, Wayne State University School of Medicine, Detroit, Michigan, United States of America; 3 Department of Anatomy and Cell Biology, Wayne State University School of Medicine, Detroit, Michigan, United States of America; 4 Department of Center for Molecular Medicine and Genetics, Wayne State University School of Medicine, Detroit, Michigan, United States of America; 5 Program in Reproductive and Adult Endocrinology, Eunice Kennedy Shriver National Institute of Child Health and Human Development, NIH, DHHS, Bethesda, MD, United States of America; Chinese Academy of Sciences, CHINA

## Abstract

**Introduction:**

The growth factor HBEGF is upregulated post-transcriptionally in the low O_2_ environment of the human placenta during the first 10 weeks of pregnancy. We have examined the possible roles of HBEGF turnover and micro-RNA (miRNA) in its regulation by O_2_ in human first trimester trophoblast.

**Methods:**

HTR-8/SVneo trophoblast cells were cultured at 2% or 20% O_2_. The cells were transfected with a dual luciferase reporter construct (psiCHECK-2) containing no insert (control), the HBEGF 3’ untranslated region (3’UTR), or sub-regions of the 3’UTR, as well as with siRNA for DGCR8. RNA was extracted from trophoblast cells cultured at 2% O_2_ for 0–4 h for next-generation sequencing. HBEGF was quantified by ELISA. HBEGF, DGCR8, and β–actin were examined by western blotting.

**Results:**

Protein turnover studies, using 10 μg/ml cyclohexamide, 1 μg/ml lactocystin, or 100 μg/ml MG132, demonstrated faster HBEGF degradation at 20% O_2_ than 2% O_2_, mediated by the proteasome. However, proteasome inhibition failed to initiate HBEGF accumulation at 20% O_2_. Reporter assays, comparing to empty vector, demonstrated that the intact HBEGF 3’ UTR inhibited expression (0.26), while fragments containing only its flanking regions increased reporter activity (3.15; 3.43). No differential expression of miRNAs was found in trophoblast cells cultured at 2% and 20% O_2_. Nevertheless, HBEGF upregulation at 2% O_2_ was blocked when the miRNA-processing protein DGCR8 was silenced, suggesting a role for miRNA.

**Conclusion:**

Our findings suggest involvement of flanking regions of the 3’UTR in activating HBEGF protein synthesis in response to 2% O_2_, possibly through a miRNA-mediated mechanism.

## Introduction

**Fig 1:**
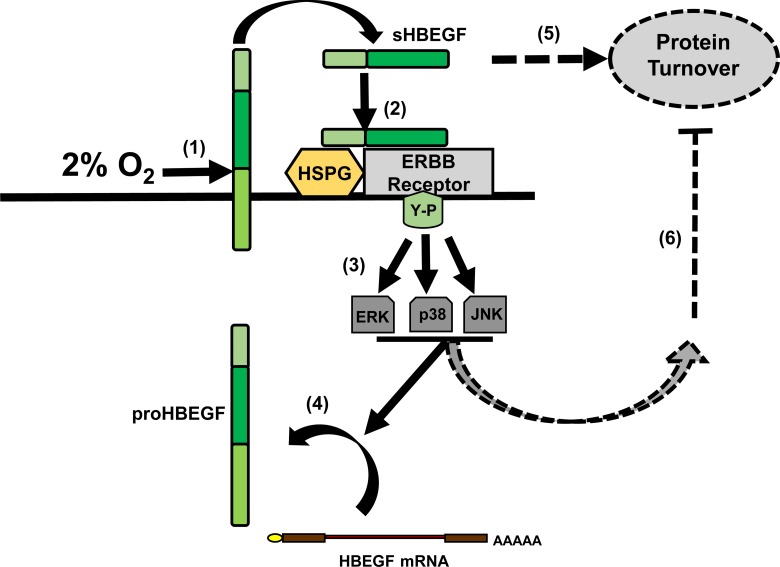
Regulation of HBEGF by downstream signaling in human TB cells. Low (2%) O_2_ induces cleavage of the extracellular domain of proHBEGF (1). The released sHBEGF binds to ERBB receptors through its EGF-like domain and to heparan sulfate proteoglycans (HSPG) through its heparin-binding domain (2). Subsequent transphosphorylation of HER cytoplasmic domains at key tyrosine (Y-P) residues (3) initiates MAPK (ERK, p38, JNK) signaling (4) that increases HBEGF accumulation and inhibits apoptosis. This positive feedback loop achieves extracellular HBEGF levels sufficient to maintain TB cell survival at 2% O_2_. Alternatively, HBEGF protein turnover (5) could be inhibited by downstream MAPKs signaling (6), to induce protein accumulation.

The epidermal growth factor (EGF) family member, heparin-binding EGF-like growth factor, (HBEGF), is present in the uterus at the time of embryo implantation [1, 2], and its expression in trophoblast (TB) cells of the placenta indicate its central role in early implantation and subsequent placentation [2]. Since, the placenta is not fully oxygenated until after the 10^th^ week of pregnancy, placentation proceeds in a low O_2_ (2%) environment. *In vitro* work has demonstrated that HBEGF protein levels are upregulated in a human first trimester TB cell line at low O_2_ (2%) [3, 4]. Furthermore, HBEGF protects TB cells from apoptosis, and promotes their invasion. Preeclampsia, in which TB invasion is reduced [5, 6] and apoptosis elevated [7–9], is characterized by reduced expression of HBEGF and other components of EGF signaling system in TB cells [10, 11]. A mechanism for the regulation of HBEGF by O_2_ in TB cells is beginning to emerge (Fig 1), based on recent studies of a first trimester TB cell line. Transmembrane HBEGF cleavage in response to low O_2_ initiates autocrine signaling that increases HBEGF levels to a concentration that inhibits apoptosis [3]. The autocrine upregulation of HBEGF requires activation of its cognate receptors (ERBB1 and ERBB4), and downstream signaling through any one of three MAPK pathways initiated by MAPK 1/3, MAPK14 or MAPK8 [12]. Previous work has demonstrated that HBEGF is not transcriptionally regulated by O_2_, although its protein levels increase by over 100-fold at low O_2_ [3]. HBEGF mRNA levels are high and remain unchanged by exposure to low O_2_. It remains to be determined at what point downstream of HBEGF shedding HBEGF accumulation is regulated.

Regulation of transcription and translation can be disparate processes, depending on the gene and cellular context [7, 13]. The abundance of specific proteins can be regulated post-translationally by altering their turnover rates [14]. Proteins with fast turnover rates are generally characterized as low abundance [15, 16], intrinsically unstructured [17, 18], or aggregation prone [19, 20], and are mostly involved in signal transduction and transcriptional activation [16, 21, 22]. It can be hypothesized that HBEGF downstream signaling impedes its protein turnover rate to initiate its accumulation through autocrine signaling (Fig 1). Certain proteins can be regulated by either stabilization [23] or destabilization [24, 25] of their mRNA. A prior study has shown that HBEGF mRNA is stabilized in HeLa cells exposed to a chemotherapeutic agent [26]. Because HBEGF transcript levels are not altered by O_2_ fluctuation [3], an alternate hypothesis is that mRNA translation is a factor in its regulation. There is evidence that microRNAs (miRNA) could directly or indirectly regulate protein expression by increasing translational rates [27, 28].

MicroRNAs are ~22 nt endogenous small RNA species that primarily target the 3’ untranslated region (3’UTR) of mRNA transcripts, and are well known for their roles in suppressing translation or inducing degradation of mRNA [29, 30]. DGCR8 is a gene within the “DiGeorge syndrome chromosomal region (DGCR)” at human chromosome 22q11 [31]. DGCR8 combines with Drosha to form a stable microprocessor, or pri-miRNA processing, complex [32–34]. MicroRNAs are transcribed as primary miRNA transcripts in the nucleus by RNA polymerase II, and are subsequently processed by the microprocessor complex into shorter pre-miRNA hairpin precursors. The pre-miRNAs are exported into the cytoplasm and processed into their mature ~22 nt form by dicer [35]. Mature miRNAs are incorporated into RNA-induced silencing complexes (RISCs) or miRNPs (miRNA ribonucleoproteins) where miRNAs target mRNAs by binding with perfect complementarity to a conserved 6–8 nt seed region in the 3’UTR. MicroRNAs have the unique capacity to bind a specific target transcript and, due to imperfect base pairing at nucleotides 10–11, inhibit translation without inducing degradation of the mRNA [30, 35, 36]. A miRNA-mediated mechanism could be responsible for the translational regulation of HBEGF by autocrine signaling.

Specific miRNAs are expressed in placental tissue [37–39] and TB cells [40, 41]. When introduced into cariocarcinoma cell cultures, miRNAs target functional proteins. Mir-152 targets HLA-G in JEG3 cells [42], miR-34a targets Notch1 and Jagged1 in HeLa and JAR cells [43], and miR-199b targets SET (protein phosphatase 2A inhibitor) in BeWo and JAR cells [44]. Donker et al. [41] demonstrated in primary TB from term placenta that the miRNP machinery is present in cells cultured for 24–72 h at 20% or < 1% O_2_, and silences MED1 (Mediator complex subunit 1) expression through differential regulation of miRNAs [45]. Regulated expression of miRNAs that target HBEGF mRNA could likewise mediate its translational regulation downstream of HBEGF shedding.

In this study, we have examined whether the regulation of HBEGF protein is mediated by its turnover rate, or if other post-transcriptional mechanisms are operative. Specifically, we examined reporter expression under control of the 3’UTR of HBEGF, the role of differential miRNA expression in regulating HBEGF translation, and the effect of global miRNA silencing by DGCR8 knockdown. Our findings suggest a less commonly reported role for miRNA, and presumably the RISC, in enabling translation from constitutively expressed HBEGF message downstream of HBEGF signaling induced at 2% O_2_.

## Materials and Methods

### Cell Culture and Treatments

The first trimester human cytotrophoblast cell line HTR-8/SVneo [46] was cultured at either 20% O_2_ or 2% O_2_, as previously described [47, 48]. Cells were cultured for the indicated times in 10 μg/ml cyclohexamide to block *de novo* translation (Sigma-Aldrich), 1 μg/ml lactocystin or 100 μg/ml MG132 to inhibit the proteasome (EMD Biosciences). The HTR-8/SVneo cell line retains important characteristics of primary cultures of human first trimester cytotrophoblast cells [49], making it a suitable model for this study. This cell line expresses the TB-specific marker KRT7. When grown on Matrigel, HTR-8/SVneo cells expresses the TB specific marker, HLA-G, and expression of integrin alpha 1 is upregulated, while alpha 6 is downregulated (integrin switching). The expression of KRT7, β-hCG and HLA-G, as well as lack of vimentin, is routinely checked, and cells are maintained between passages 30–50.

### Western Blotting

Western blots were performed as previously described [49]. Cellular lysates were lysed in SDS sample buffer containing 5% β-mercaptoethanol, run on precast 4%-20% Tris-HCl gradient gels (BioRad), and transferred to nitrocellulose membranes. Monoclonal mouse antibodies against DGCR8 (Proteintech) and GAPDH (Ambion) were diluted in 5% milk dissolved in TTBS to 0.3 μg/ml and 1 μg/ml, respectively. A polyclonal antibody against HBEGF (R&D Systems) was diluted to 0.2 μg/ml in 5 mg/ml BSA in TTBS.

### ELISA

The HBEGF DuoSet ELISA Development kit (R&D systems) was used, as previously described [3, 48]. The optical density of the final reaction product was determined at 450 nm, using a programmable multiplate spectrophotometer (Power Wave Workstation; Bio-Tek Instruments) with automatic wavelength correction.

### Generation of 3’UTR Luciferase Reporter Vectors

Regions of the HBEGF 3’UTR were amplified by PCR. Restriction sites for XhoI and NotI were added to forward and reverse primers (IDT DNA), respectively. 3’UTR regions amplified by the primers and the expected amplicon sizes are listed in Table 1, The PCR amplified 3’UTR regions were cloned into the psiCheck-2 vector (Promega), in which two luciferase genes (Firefly and Renilla) driven by separate promoters are present. HBEGF 3’UTR inserts were cloned into a region immediately following the Renilla luciferase gene, which serves as the reporter, while the Firefly luciferase gene is an internal control. After isolating RNA from TB cells (miRNeasy, Qiagen), cDNA was generated using the Omniscript Reverse Transcription kit (Qiagen). HBEGF 3’UTR fragments were amplified by endpoint PCR, using HotstarTaq Plus Master Mix kit (Qiagen), according to the manufacturer’s instructions. In a total reaction volume of 50 μl, 0.5 μM of each primer and 50 ng of cDNA were combined. PCR products were processed using Wizard SV Gel and PCR Clean-up Kit (Promega). Cleaned amplicons and the PsiCheck2 vector (1 μg each) were digested with both XhoI and NotI restriction enzymes (Promega) for 1 h at 37°C in a thermal cycler, followed by a 15 min 70°C inactivation step. Amplified 3’UTR fragments and PsiCheck2 vector were combined and ligated, using Promega’s LigaFast Rapid DNA Ligation System. To verify ligation, 1 μl of ligated product was amplified by PCR, using primers that bind outside of the PsiCheck2 vector’s multiple restriction site, as indicated in Table 1.

**Table 1:** Primer sequence for amplification of HBEGF 3’UTR and vector sequencing.

Forward Primers	Sequence	Target Site on HBEGF mRNA
HBEGF 3’UTR	AAACTCGAGGGAGGTTATGATGTGGGAAAATGA	844
HBEGF 3’UTR	AAACTCGAGCCTTTGCCACAAAGCTAGGA	1644
HBEGF 3’UTR	AAACTCGAGTTGCCTAGGCGATTTTGTCT	1829
HBEGF 3’UTR	AAACTCGAGAACAGGGAACATTGGAGCTG	2137
PsiCheck-2 vector	AGGACGCTCCAGATGAAATG	
Reverse Primers	Sequence	Target Site on HBEGF mRNA
HBEGF 3’UTR	AAAGCGGCCGCAGATCCCTTGGTGGTACT	1363
HBEGF 3’UTR	AAAGCGGCCGCCAGGAAATTGCCAAAGTA	1603
HBEGF 3’UTR	AAAGCGGCCGCCCAAGTTAACCCCTACATCCTG	1778
HBEGF 3’UTR	AAAGCGGCCGCATGAACCAGGTTTGGAAATACA	2315
PsiCheck-2 vector	CAAACCCTAACCACCGCTTA	

### Bacterial Transformations

One microliter of the ligation reaction mixture or empty PsiCheck2 vector was placed on ice, combined with 50 μl of JM109 bacteria (Promega) and heat shocked. Bacteria were incubated on an LB Agar plate containing AMP-100, X-GAL-80, IPTG-50 (Teknova) overnight at 37°C. Colonies were grown and expanded in 3 ml of LB broth (Becton, Dickinson & Co.) containing 100 μg/ml of Ampicillin. Bacterial lysates were prepared and amplified by PCR with primers in Table 1 to select colonies that displayed a correctly-sized insert. Vectors were isolated using the Wizard Plus SV Miniprep kits (Promega), and 100 ng were used for transfection reactions. Vectors were sequenced using the same primers utilized to verify ligation, and aligned to the HBEGF mRNA sequence (PUBMED sequence NM_0001945.2) using Geneious [50].

### Transfection and Luciferase Assay

HTR-8/SVneo cells were grown to 75% confluence and cultured for an additional 24 hr in 1 ml serum-free media (DMEM/F-12 with 5 mg/ml BSA) containing 3 μl of FuGene-6 and 1 μl of vector (100 ng). The cells were lysed in 500 μl 1X passive lysis buffer (Promega). Lysates (20 μl) was used in each dual luciferase reaction, conducted according to the manufacturer’s protocol (Dual Luciferase Reporter Kit, Promega). Negative controls containing untransfected cells were included. Each sample received 100 μl of LARII reagent, and was read after adding 100 μl Stop & Glo reagent (Promega) using a Veritas microplate luminometer (Turner Biosystems). Renilla measurements were normalized to Firefly measurements. Each transfection experiment was repeated at least 3 times.

### Library Preparation for NGS

TB cells maintained at 20% O_2_ were cultured at 2% O_2_ for 0–4 hrs and RNA was collected. For RNA-seq, both long RNA and small RNA (sRNA) were isolated with the miRNeasy mini kit (Qiagen). The sRNA fraction was quantified and its purity assessed, using an Agilent 2100 Electrophoresis Microfluidics Analyzer. A sRNA digital gene expression library was constructed by converting RNA into an adapter-ligated cDNA library, using a ScriptSeq Miner (Epicentre) and following the manufacturer’s protocol. Each cDNA sample was bar-coded and the resulting 12 cDNA libraries (3 replicates for each O_2_ concentration) were combined for sequencing on Illumina’s Genome Analyzer II sequencer at the Wayne State University Applied Genomics Technology Center.

### Sequencing Data analysis

Demultiplexing was performed using configure BclToFastq.pl script (CASAVA-1.8.2; Illumina Inc.) with default parameters for quality and index identification. The sequencing data was aligned to the human genome build (HG19) and to the ribosomal sequences 18s and 28s using Genomatix mapping station (GMS). The GMS generated tsv.files were exported to the Genomatix genome analyzer and subjected to the miRNA expression analysis tool to identify differentially expressed miRNAs.

### DGCR8 Knockdown

HTR-8/SVneo cells were tested for toxicity due to NeoFX transfection reagent (Ambion), using the Multitox-Fluor Multiplex Cytoxocity Assay Kit (Promega) to determine an optimal concentration for transfection, which was 0.75 μl of NeoFX reagent per well in a 96-well plate, (<10% toxicity after 48 hrs). Cells were transfected in a 96-well plate (5,000 per well) for 48 hr with various concentrations of three siRNAs (s29061, s29062, s29063; Life Technologies) that target DGCR8. Controls included no transfection, treatment with transfection agent only, transfection with a negative control siRNA and a GAPDH siRNA. Cells were fixed with ice-cold methanol for 10 min, permeabilized with 0.1% Triton-X100 for 15 min, stained overnight with primary antibody against DGCR8 (Santa Cruz Biotechnology, Inc.) at 4°C, incubated for 1 hr at room temperature with an anti-mouse/anti-rabbit labeled polymer (DAKO EnVision Dual Link), and visualized with DAB. Based on preliminary experiments, 50 nM siRNA was chosen for DGCR8 knock down.

### qPCR

RNA from HTR-8/SVneo cells was collected using the miRNeasy kit (Qiagen), according to the manufacturer’s protocol. RNA concentration was determined using the NanoDrop spectrophotometer and purity was ascertained with a microfluidic Bioanalyzer (Agilent Technologies—2100 Electrophoresis Bioanalyzer Instrument). RNA was used in subsequent qPCR [51] analysis. Reverse transcription was performed using the Quantitect Reverse Transcription kit (Qiagen), and qPCR for HBEGF was conducted with the Quantitect SYBR Green PCR kit without UNG (Qiagen), in a final volume of 25 μl. GAPDH was used as a housekeeping gene to normalize the data. Semi-quantiative analysis was performed according to the ΔΔCt method [52]. Primers for GAPDH and HBEGF were obtained from Qiagen.

### Statistics

All experiments were repeated at least 3 times and reported as mean ± SEM. Statistical significance was determined at *p*<0.05 by ANOVA, Student-Newman-Keuls tests and Dunnett t-test using SPSS version 12.0 statistics software (SPSS).

## Results

### HBEGF regulation by protein degradation

**Fig 2:**
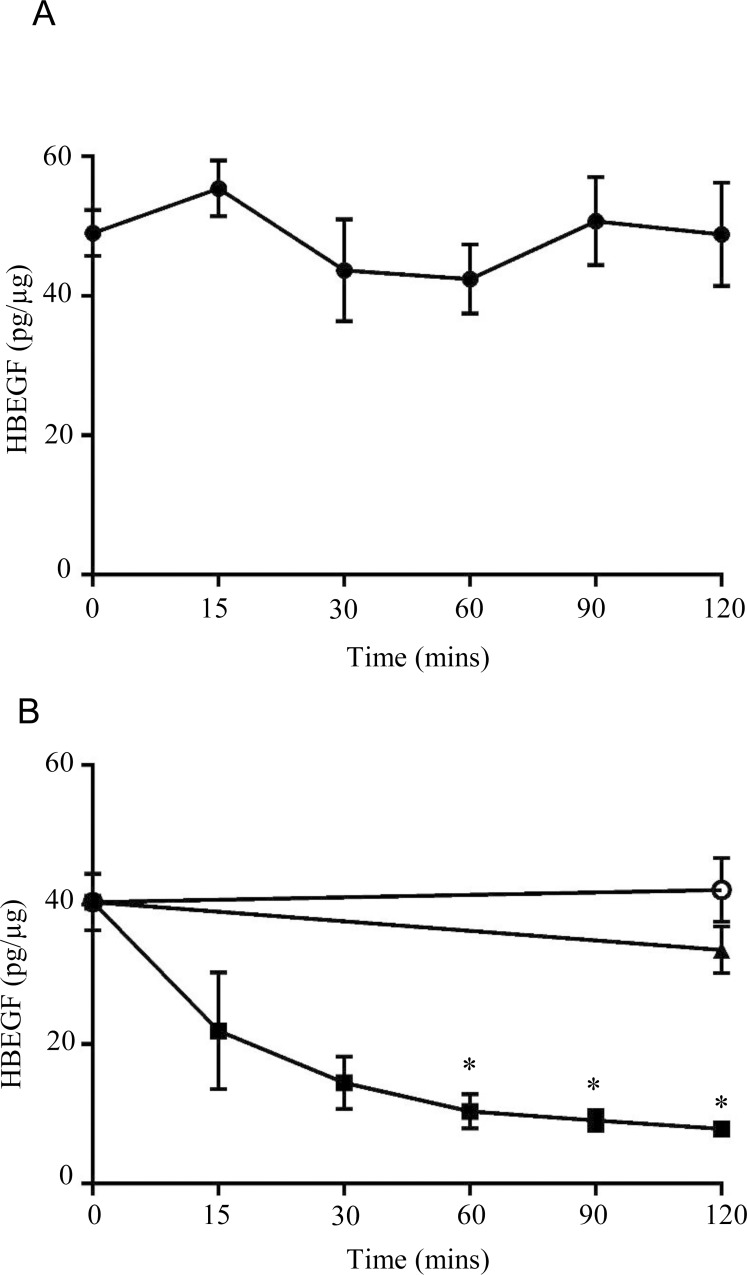
Effect of O_2_ concentration on HBEGF stability. HTR-8S/Vneo cells were cultured for 6 h at 2% O_2_, allowing HBEGF protein to accumulate. Afterwards, cells were cultured 2 additional hours (A) at 2% O_2_ while exposed to cyclohexamide, or (B) at 20% O_2_ in the absence (black square) or presence of either 1 μg/ml lactocystin (white circle) or 100 μg/ml MG132 (triangle). Cell lysates were collected at the indicated times and HBEGF protein levels quantified by ELISA. * p < 0.05 according to ANOVA and Student-Newman-Keuls t-tests.

The observed increase in HBEGF protein levels at low O_2_ could reflect a decrease in protein turnover rate at 2% O_2_. HBEGF protein levels were elevated by culture at 2% O_2_ for 6 h and cultured for an additional 2 hours with cyclohexamide. HBEGF levels remained unchanged (Fig 2A), indicating a very low turnover rate. In contrast, cells shifted to 20% O_2_ after 6 h at 2% O_2_ showed a marked destabilization of HBEGF protein, which decreases significantly within 15 min (Fig 2B). HBEGF degradation was abrogated if cells were concomitantly treated with the proteasome inhibitors MG132 or lactocystin after shifting cells to 20% O_2_, suggesting that HBEGF turnover requires the proteasome. However, HBEGF protein did not increase when cells cultured at 20% O_2_ were treated with proteasome inhibitors (data not shown), suggesting that stabilization of HBEGF degradation is insufficient for its accumulation at 20% O_2_, and that its accumulation at 2% O_2_ requires activation of a translational mechanism. These finding further indicate that destabilization by proteolysis through the proteasome clears HBEGF during reoxygenation.

### Regulation by the HBEGF 3’UTR

**Fig 3:**
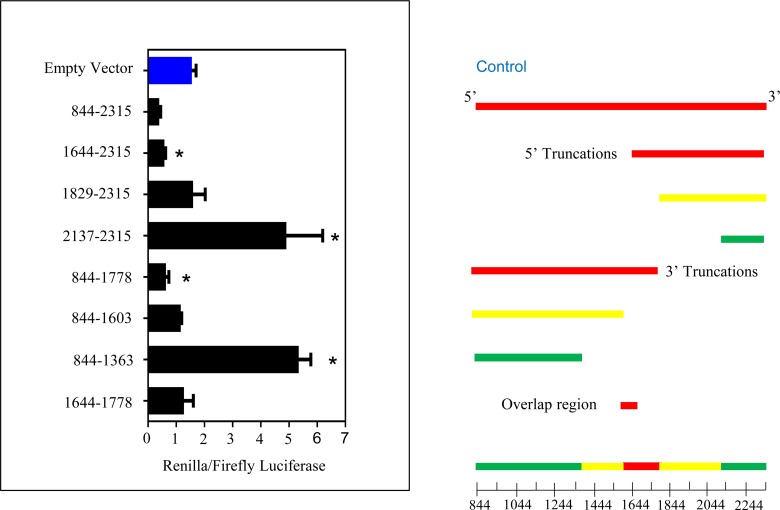
Translational regulation by HBEGF 3’UTR. Dual luciferase assays used constructs containing the indicated sequences of the HBEGF mRNA inserted 3’ to Renilla luciferase. The left panel shows activities of the Renilla luciferase normalized to a constitutive firefly luciferase. N = 3. *, p<0.05 vs empty vector control (blue bar), ANOVA with Dunnett t-test. Right panel shows the locations of the inserts in colors indicating their effect on translation; yellow, neutral; red, suppression; green, activation. A scale in base pairs is shown below.

The differential regulation of HBEGF synthesis by O_2_ in human TB cells could be the result of translational suppression at 20% O_2_, or activation of protein synthesis from its mRNA at 2% O_2_ under the direction of its large (1455bp) 3’UTR, a region of mRNA known to be involved in transcript stability [53]. To determine whether the 3’UTR of HBEGF can control translation, a PsiCheck-2 luciferase reporter with 3’ inserts of the entire HBEGF 3’UTR or its subdomains was prepared and transfected into TB cells cultured at 20% O_2_. The presence of certain portions of the HBEGF 3’UTR altered Renilla Luciferase production, as compared to an empty vector control (Fig 3) A vector containing the full-length 3’UTR (844–2315) significantly (p = 0.008) repressed reporter activity, as did vectors containing the 5’ subdomain (844–1778; p = 0.031) or 3’ subdomain (1644–2315; p = 0.019). In contrast, the 134 bp region overlapped by the two larger domains did not regulate reporter activity. Vectors containing regions 844–1603, 1644–1778, and 1829–2315 were also not active. Vectors containing either the 5’ (844–1363) or 3’ (2137–2315) flanking regions of the 3’UTR upregulated Renilla luciferase activity at both 20% O_2_ and 2% O_2_ (p<0.001 for both). The vectors containing the two flanking regions more significantly upregulated reporter at 20% O_2_ as compared to 2% O_2_ (t-test, p = 0.022 and p<0.001, respectively). Based on these observations, it appears that the two flanking domains of the 3’UTR increase translational activity in isolation from the central 3’UTR regions, while the presence of regions from 1363bp-1644bp and 1778bp-1829bp instigated translational suppression. These data are consistent with two opposing activities within the 3’UTR that respectively activate and suppress translation from the HBEGF transcript.

### Next generation sequencing to identify differentially expressed miRNAs

**Fig 4:**
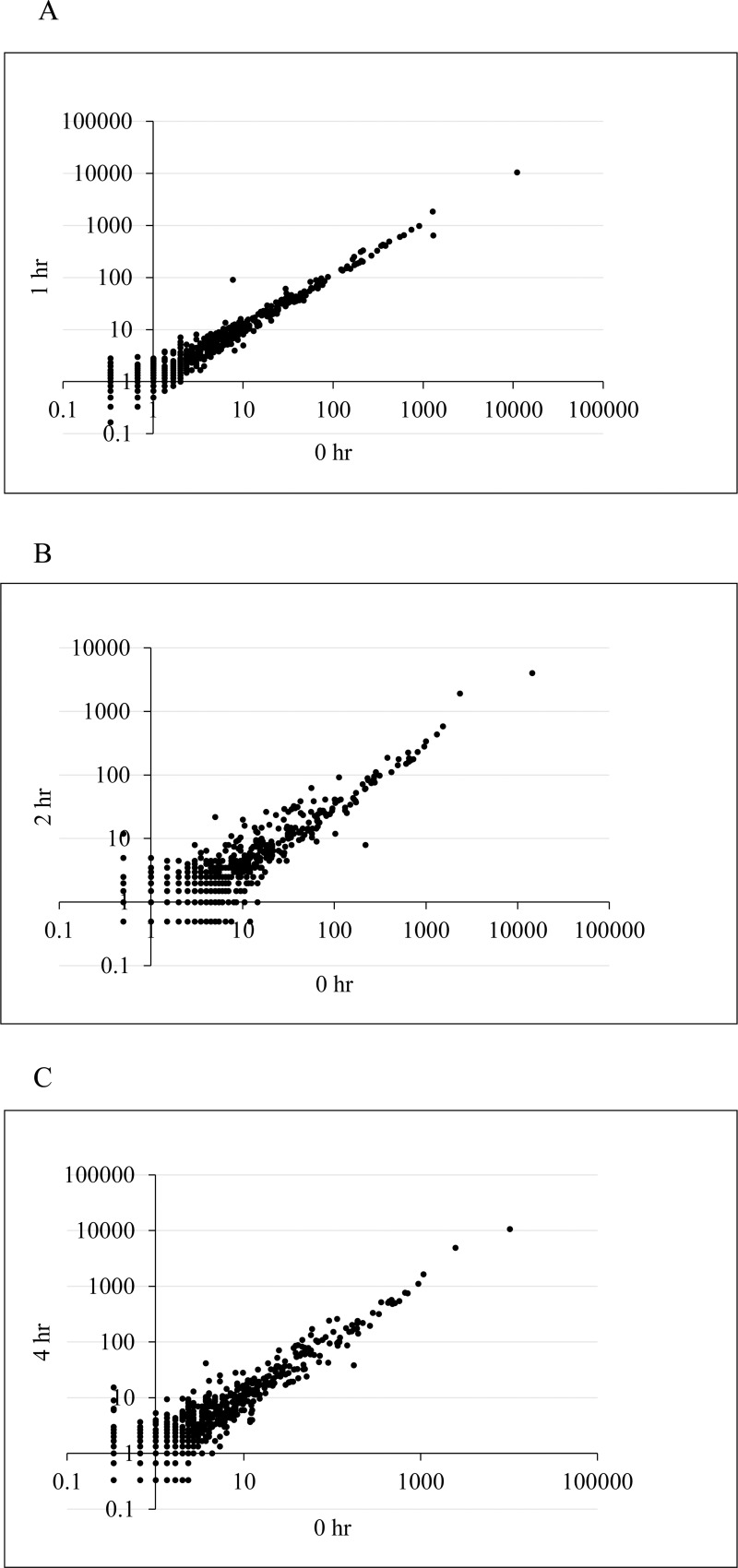
Effect of O_2_ on miRNA expression by next generation sequencing: Log-log comparisons of RPKM values for 2556 miRNAs in HTR8/SVneo cells after transfer from 20% O_2_ to 2% O_2_ for 0 h (x-axis, A-C), 1 h (A, y-axis), 2 h (B, y-axis) or 4 h (C, y-axis). No miRNAs deviated significantly from a straight line with slope of 1.

To determine if HBEGF translation is regulated in response to O_2_ by differentially expressed miRNAs that interact with its 3’UTR, expression of miRNAs was examined globally by NGS. TB cells cultured at 20% O_2_ were exposed to 2% O_2_ for 0, 1, 2 and 4 h, and barcoded libraries were constructed for NGS. The software analyzed a total of 2556 miRNAs based on the HG19. A log-log plot of RPKM values comparing 0 h to later time points (Fig 4), and statistical analysis by the Genomatix genome analyzer software, revealed that no miRNAs were differentially expressed. These findings suggest that if a mechanism involving miRNAs regulates HBEGF translation, it does not do so through changes in the expression of the miRNAs.

### Global inhibition of miRNA processing

**Fig 5:**
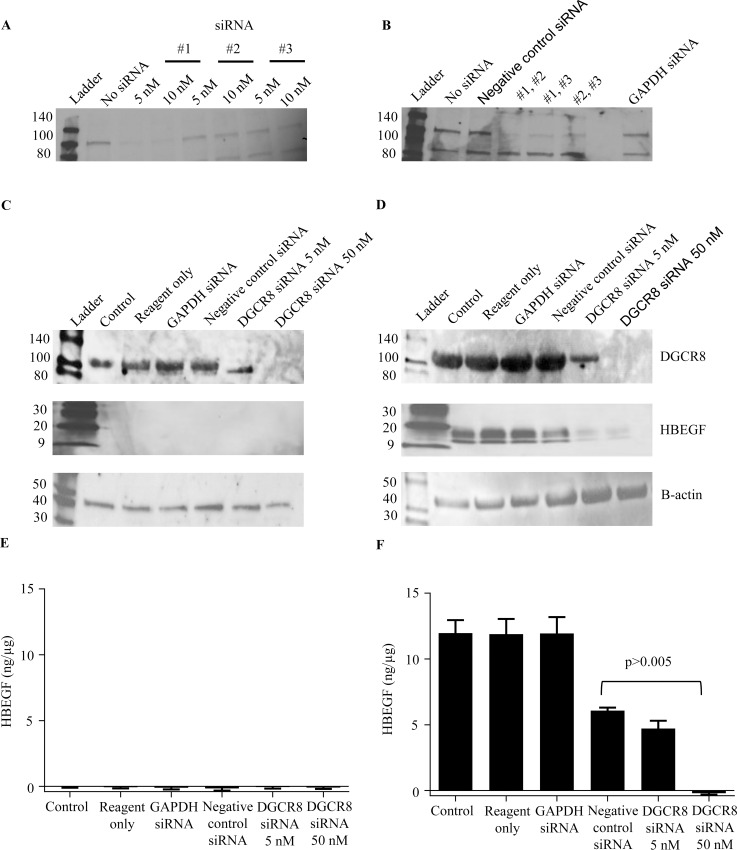
Effect of DGCR8 Knockdown on regulation of HBEGF by O_2_. Western blots of DGCR8 in lysates of HTR-8/SVneo cells treated for 48 hr with (A) 5 nM or 10 nM of three different DGCR8 siRNA and (B) different pairings of the three siRNA (5nM). C-F. DGCR8 (~120kDa), HBEGF (~22kDa) and β-actin (~45kDa) were assessed in lysates of HTR-8/SVneo cells (control), cells treated with transfection reagent only, or cells transfected with GAPDH siRNA, 50 nM negative control siRNA, and 5 nM and 50 nM DGCR8 siRNA. After 48 hrs, culture was continued for 4 hrs at 20% O_2_ (C, E) or 2% O_2_ (D, F). Lysates were assayed by western blot (C, D) or an ELISA for HBEGF (E, F). Untreated trophoblast cells and trophoblast cells containing transfection reagent alone served as Control. *, p<0.005, compared to Control.

We further examined the potential role of miRNA in regulating HBEGF, using siRNA to silence DGCR8, which should globally suppress miRNA processing and deplete all miRNA [34]. DGCR8 was knocked down in HTR-8/SVneo cells by transfection for 48 h with siRNA. Controls included no transfection, treatment with transfection reagents only, and transfection with either a negative control siRNA or a GAPDH siRNA. Culture was either continued for 4 h at either 20% O_2_, or shifted to 2% O_2_. Western blots for DGCR8 and toxicity assays were used to optimize conditions for knockdown (Fig 5A and 5B). DGCR8 was greatly reduced in cells transfected with 50 nM of the targeting siRNA, but not in control treatments. Unexpectedly, HBEGF expression did not increase when DGCR8 was knocked down at 20% O_2_ (Fig 5C), suggesting that HBEGF expression was not repressed by miRNA at high O_2_. Repression of translation by miRNA often occurs through degradation of message [54]. However, HBEGF mRNA was unchanged after DGCR8 knockdown and unaffected by O_2_ concentration (Table 2). Surprisingly, the upregulation of HBEGF at 2% O_2_ was inhibited in the DGCR8 knockdown compared to controls (Fig 5D). These results were verified by ELISA to quantify HBEGF, which demonstrated levels near 0.01 pg/μg in all treatments at 20% O_2_ (Fig 5E). At 2% O_2_, HBEGF levels rose to 5 pg/μg (range 4.2–6.4) in all controls, while cells transfected with 50 nM DGCR8 siRNA contained only 0.88 ± 0.22 pg/μg HBEGF (Fig 5F). These findings indicate that HBEGF expression was not repressed by miRNA at high O_2_, and further suggest that miRNA is required for increased translation of HBEGF at 2% O_2_.

**Table 2:** HBEGF mRNA in HTR-8/SVneo cells with or without (Control) exposure to DGCR8 siRNA measured by qPCR*.

20% O_2_		2% O_2_	
Control	DGCR8 siRNA 50nM	Control	DGCR8 siRNA 50nM
1.3 ± 0.26	0.92 ± 0.27	1.07 ± 0.30	1.39 ± 0.39

*Relative expression of HBEGF mRNA normalized to GAPDH mRNA. Mean ± SE, n = 3.

## Discussion

The present study demonstrates the complexity of HBEGF upregulation, and suggests the potential role of miRNAs. Blocking HBEGF degradation with proteasome inhibitors failed to instigate accumulation of HBEGF in TB cells cultured at 20% O_2_, suggesting that the upregulation of HBEGF at low O_2_ is not due solely to decreased HBEGF turnover, but requires increased translation of abundant HBEGF mRNA. HBEGF turnover was indeed strikingly faster at 20% than at 2% O_2_, which could facilitate clearance of the protein during reoxygenation.

Given the lack of transcriptional regulation [3], it was hypothesized that HBEGF could be regulated translationally by its large (1461 bp) 3’UTR. Luciferase reporters have been used to assess translational regulation by 3’UTRs [55] and provided evidence here that cloned regions of the HBEGF 3’UTR regulate its translation with domain-specific stimulatory or inhibitory activities. Others have examined translational suppression by miRNA with cloned 3’UTR regions by transfecting with an additional vector that contains target miRNA in HEK293 cells [56] and HTR-8/SVneo cells [45]. The methodology that we chose is advantageous because it more closely recapitulates *in vivo* conditions by relying upon endogenous miRNA and translational machinery. Although there was no effect of changing O_2_ concentrations, the flanking regions of the 3’UTR (844–1363 and 2317–2315) strongly upregulated luciferase activity, suggesting that these domains could have a role in elevating HBEGF translation at 2% O_2_. In contrast, regions towards the center of the 3’UTR (1363–1603 and 1778–2315) repressed luciferase production, and are likely responsible for the overall repressive effect of the complete 3’UTR sequence. Luciferase assays showed that discrete regions of the 3’UTR are sufficient to differentially regulate HBEGF.

An unbiased NGS approach failed to find miRNAs that are differentially expressed at 20% and 2% O_2_. Therefore, HBEGF was apparently not regulated by changes in the levels of miRNAs that interact with its transcript, but other components of the RISC interacting with miRNAs could be involved. The requirement for miRNA was further examined by blocking miRNA synthesis in a DGCR8 knockdown experiment, realizing that it could interfere with other unrelated critical cellular processes. Suh et. al used Dgcr8 knockout mice to distinguish the effects of miRNA and endogenous siRNA during oocyte maturation and preimplantation development, comparing the effects on development and mRNA expression to oocytes and embryos deficient in Dicer [57]. During treatment with DGCR8 siRNA, the low expression of HBEGF at 20% O_2_ persisted. However, HBEGF protein failed to increase at 2% O_2_, suggesting that miRNA is linked to increased HBEGF translation in either a positive or indirect manor at 2% O_2_ rather than through suppression at 20% O_2_. This is consistent with the observation that HBEGF mRNA expression was unchanged by DGCR8 knockdown at either O_2_ concentration. Hence, miRNA could be responsible for facilitating the upregulation of HBEGF through changes in other components of the RISC at low O_2_ that increase the influence of the flanking regions of the 3’UTR (844–1363 and 2137–2315), or reduce the influence of the repressive central domains. The regulatory activity of the RISC can be affected by structural changes in the 3’UTR, such as shortening. For example, 3’ UTR shortening can occur due to cleavage of the alternative poly(A) sites on the 3’UTR of mRNA, and this can lead to changes in protein translation efficiency, mRNA stability, or mRNA localization [58].

Alternatively, miRNAs or other regulatory factors that bind regions outside the 3’UTR (for example, to the 5’UTR) could accelerate translation of HBEGF at 2% O_2_. There is evidence that miR-10a binds to the 5’oligopyrimidine tract motif in the 5’UTR of ribosomal proteins in neural U87 cells and mediates translational upregulation [59]. MiR-122 targets the 5’non-coding region of the hepatitis C viral genome to positively regulate accumulation of viral RNA [60] through requisite occupation of two miR-122 binding sites in hepatic cells [61]. Hence, miRNA or other regulatory factors that bind regions outside the 3’UTR could integrate with regions in the 3’UTR to induce translation at 2% O_2_.

In the absence of a change in miRNA profile, HBEGF translation could be controlled by other components of the RISC, including regulatory proteins activated downstream of MAPK signaling, which is required for HBEGF upregulation at 2% O_2_ [12]. Iwasaki et. al found that loading of sRNA duplexes into the RISC complex requires HSP70/HSP90 chaperones activity [62]. We find in TB cells that HSP70 transcription is induced at low O_2_ (Jain et al., unpublished). Alternatively, there is evidence that the stability of AGO2 in the RISC is enhanced by EGFR/ERBB1 through MAPK signaling in cancer cell line MDA-MB-231 [63]. EGFR induces phosphorylation of Y393 in AGO2 to inhibit miRNA maturation, leading to tumor cell survival [64]. Using mass spectrometry, multiple phosphorylation sites have been identified in AGO2 from HEK293 cells [65]. Phosphorylation of Y529 in the RNA-binding pocket results in inefficient binding of sRNA to the protein, suggesting a mechanism for the alternate binding and release of miRNAs. Additionally, studies in Hela cells demonstrate that phosphorylation of Ago2 by AKT3 and MK2 at S387 upregulates miRNA-mediated translational repression of endogenous miRNA targets [66]. Some RNAs can displace miRNAs from their target mRNAs. An example is competing endogenous RNA (ceRNA) [67] or the recently described circular RNA (circRNA) [68], where the RNAs not only manifest themselves in the sequestration of miRNAs, but also act through the direct or indirect binding of RNA-binding proteins.

Genes other than HBEGF are also translationally regulated by O_2_. For example, low O_2_ increases ATF4 protein, but doesn’t affect its mRNA, in hippocampal neurons [69]. In addition, chemically-induced endoplasmic reticulum (ER) stress, comparable to effect of low O_2_, shifts ATF4 mRNA from monoribosomes to polyribosomes, indicating that its preexistent mRNA pool is inefficiently translated until cells experience ER stress. This shift to polyribosomes was dependent on the presence of ER-resident eIF2alpha kinase/PERK, suggesting that eukaryotic initiation factor 2A (EIF2A) is phosphorylated prior to translation of ATF4 [70].

Additional studies are necessary to better understand the mechanism controlling HBEGF autocrine upregulation. We have presented evidence that the RISC is a key site of regulation; therefore, a proteomic analysis of the RISC could be used to identify components that are altered in response to HBEGF downstream signaling. The significance of this mechanism *in vivo* is unclear, because this investigation was conducted in a trophoblast cell line. However, we have found that HBEGF is indeed upregulated at low O_2_ in villous explants (manuscript submitted). The development of an animal model to experimentally investigate the proposed mechanism would be useful for further understanding of HBEGF regulation by O_2_ *in vivo*.

The present study demonstrates that HBEGF is post-transcriptionally regulated by O_2_ through a mechanism involving interactions of miRNAs with its 3'UTR. Luciferase assays identified discrete regions of the 3’UTR that could differentially regulate HBEGF translation. A role for miRNA was suggested by knocking down the miRNA-processing protein, DGCR8. Although no differentially expressed miRNAs were found, it can be speculated from the DGCR8 knockdown studies that miRNAs are required for translation of HBEGF, but not for its repression at 20% O_2_. The regulation of HBEGF through a targeted mechanism that increases its translation rate, putatively involving miRNA and the RISC, appears to be uncommon based on a paucity of similar reports in the literature. The elucidation of this complex regulatory mechanism could provide important insights into TB survival during early placentation, and its disruption in pregnancies with developmental pathologies.
